# Cytokine synthesis of Th-1 and Th-2 cells by atopic patients with food allergy and food intolerance

**DOI:** 10.1186/2045-7022-1-S1-P63

**Published:** 2011-08-12

**Authors:** Olga Sidorovich, Ludmila Luss, Margarita Nikonova, Almira Donetskova

**Affiliations:** 1 NRC Institute of Immunology FMBA, Out-patient Department, Moscow, Russian Federation; 2 NRC Institute of Immunology FMBA, Moscow, Russian Federation

## Background

In clinical practice we often face serious food allergy diagnostic and therapy problems because there are no specific clinical markers of allergic reactions to food. The problem is that adverse reactions to food could be caused by different mechanisms, for example sensibilisation to food allergens and food additives by food allergy. Besides, adverse reactions to food develop by different gastrointestinal tract pathologies. The algorithm of diagnostics, treatment and prevention of food allergy and food intolerance require different methodical approaches.

The aim of this research was to bring out the peculiarities of Th1 and Th2-cells cytokine synthesis among allergic patients with food allergy and food intolerance.

## Methods

In this work we analysed the expression of IL-2, 2Ra, 4, 5, 10, 12a, 12b, TGF-β, INFγ, transcription factors FOXP3, T-bet and GATA-3 which were measured by 23 patients with food allergy and by 35 patients with food intolerance by PCR method. 20 patients with food intolerance without any atopy, 20 patients with pollinosis without any signs of food allergy and food intolerance and 20 apparently healthy persons were examined as the control groups.

## Results

Cytokine expression was similar in all groups. However, a high level of IL-5 expression trend was shown in the group with food allergy. A significant increased expression of GATA-3 was shown in food allergy group.

## Conclusions

mRNA expression of cytocine genes showed by patients with food allergy and food intolerance in remission is comparable with mRNA expression by apparently healthy persons. An important role of GATA-3 has been proven in Th2-response activation by patients with IgE-dependent food allergy. Thus, the results of this work show the necessity of further research activities to improve immunological methods of examination of patients with food allergy and food intolerance and development of effective diagnostics, prevention and treatment of these diseases.

